# Socio-environmental determinants of the delay in the first dental visit: results of two population-based cohort studies in Brazil

**DOI:** 10.1590/1414-431X202010161

**Published:** 2020-11-11

**Authors:** A.L.F.H. Soares, C.C.C. Ribeiro, E.B.A.F. Thomaz, R.C.S. Queiroz, C.M.C. Alves, A.A. Ferraro, A.A.M. Silva, H. Bettiol, M.A. Barbieri, M.C.P. Saraiva

**Affiliations:** 1 Departamento de Clínica Infantil, Faculdade de Odontologia de Ribeirão Preto, Universidade de São Paulo, Ribeirão Preto, SP, Brasil; 2 Departamento de Odontologia II, Universidade Federal do Maranhão, São Luís, MA, Brasil; 3 Departamento de Saúde Pública, Universidade Federal do Maranhão, São Luís, MA, Brasil; 4 Departamento de Pediatria, Faculdade de Medicina, Universidade de São Paulo, São Paulo, SP, Brasil; 5 Departamento de Puericultura e Pediatria, Faculdade de Medicina de Ribeirão Preto, Universidade de São Paulo, Ribeirão Preto, SP, Brasil

**Keywords:** First dental visit, Epidemiology, Children's oral health, Health service utilization, Cohort study

## Abstract

The objective of this study was to describe the timing of the first dental visit and investigate the association of socioeconomic and behavioral factors with dental visit delay among 10/11-year-old children from two live-birth population cohorts with extremely contrasting socioeconomic profiles. Follow-up data (2004-2005) from cohorts of Ribeirão Preto (RP) (n=790) and São Luís (SL) (n=673) were evaluated. Delay in dental visit was defined as not visiting a dentist before the age of 7. Covariates included family socioeconomic characteristics, mother-related health behavior, and child-related characteristics. Prevalence ratios with robust standard errors were estimated. In both cohorts, less than 5% of children had visited a dentist before the age of two and about 35% of them had not visited a dentist before the age of seven. Lower mother’s schooling and lack of private health insurance were associated with the delay in first dental visit for both cohorts. A small number of mother's prenatal care visits and being from a single-father family or a family without parents were only associated in the RP cohort, while having ≥4 siblings and lifetime dental pain were associated in the SL cohort. The association with dental pain probably reveals a preventive care-seeking behavior. Therefore, the percentage of delayed first dental visit of children was very high even among those with the most educated mothers. Further studies are necessary to analyze recent changes and underlying factors related to access to first dental visit after the implementation of the National Oral Health Policy in 2006.

## Introduction

The American Academy of Pediatric Dentistry (AAPD) recommends that the first dental visit should occur at the time of first tooth eruption and no later than 12 months of age (1). Regardless of the lack of empirical evidence about the best age that a child should first be taken to a dentist and the efficacy of early dental visits (2), these guidelines are based on the premise that early detection and management of oral conditions and anticipatory guidance improve the oral health and well-being of children (3).

In spite of consensus among Dental Associations about the importance of a preventive dental visit in the first year of life, there are only a few studies reporting the timing of a child's first dental visit, with conflicting results about its effects on caries prevention and costs. Among the reasons for these inconsistencies is the difficulty in defining what a preventive dental visit is. Unfortunately, most of the studies trying to address the efficiency of early dental visits are from other countries, such as a series of studies with Medicaid (USA) data from reimbursement records (4–6). The best evidence from these studies was that delaying the first dental visit was associated with future emergency visits and restorative procedures, especially for children at highest risk of dental caries. Although Medicaid studies help us to understand some aspects of the timing of preventive dental visits, they are limited to children from very low income families under the age of five who are continuously enrolled in the program (4,5). We found only a few other population studies addressing the effects of the timing of the first dental visit. Ismail and Sohn (7), in a study conducted in Canada in an area covered by universal dental insurance, observed that only 8.4% of children had visited a dentist before the age of two, with no association with lower levels of dental caries. In that study, 96.9% of children had been to a dentist by the age of five and the majority of dental visits were preventive ones (92.4%). In the United Kingdom, where dental insurance is traditionally universal as in Brazil, although most children under the age of five had already seen a dentist (94%), only 30% had a dental visit before the age of two (8). By contrast, in the United States, with a public health system only for those below the poverty line, 46.2% of children two to five years old had never visited a dentist (9).

In Brazil, a country with a public-private mixed health system where providing free healthcare for all citizens (including oral health care) is an obligation of the State, this percentage is even higher. Despite the great advances in oral health policies, especially with the National Oral Health Policy (NOHP) in Brazil (10), inequalities related to access and utilization of dental services persist (11,12). The latest 2008 Brazilian National Household Survey (13) and National Oral Health Survey (NOHS) (14) showed that 67.2 and 46.8% of six- and five-year-old children, respectively, had never been to a dentist. Although the absence of dental visits was 1.6 times more likely to be seen among children in the poorest quintile of family income, the percentage was still very high (47.3%) among the richest ones. A similar inequality ratio was observed in a population-based cohort study of five-year-old children in the south of Brazil, which also revealed that 63% of all children had never been to a dentist by that age (15). An interesting finding from that study was that the majority of dental visits for both prevention and treatment were observed among the richest families and that those who sought preventive dental visits had a lower prevalence of dental caries. Although the study addressed the reasons for dental visits, the timing of the first dental visit was restricted to five-year-old children. Considering that only about 30% of children had been taken to a dentist by the age of five, more information is needed about the timing of the first dental visit throughout childhood and associated factors. Thus, the objective of the present study was to describe the timing of the first dental visit and to explore factors associated with dental visit delay in two live-birth population cohorts with extremely contrasting socioeconomic profiles in Brazil.

## Material and Methods

This study is an analysis of the follow-up data of two live-birth population cohort studies conducted with the same methodology (16,17) by the same research team in Brazil. One cohort was started in 1994 in the city of Ribeirão Preto (RP), situated in one of the wealthiest areas of southeast Brazil (São Paulo State), with a Municipal Human Development Index (MHDI) of 0.855 in 2000 (18) and a population of 461,427 inhabitants in 1994 (19). The other cohort was established in 1997 in the city of São Luís (SL), capital of the State of Maranhão (northeast region), with an MHDI of 0.658 in 2000 (18) and with 781,068 inhabitants in 1997 (20).

The RP cohort included virtually all (99%) live births that occurred in the city from April to August 1994 and the SL cohort comprised a systematic sample of 96.3% of all live births occurring in hospitals from March 1997 to February of 1998. Losses were less than 5% in RP and 6% in SL, represented mostly by refusal to participate and anticipated discharge from the hospital. Excluding twin births, the final samples for RP and SL were 2,846 and 2,443 participants, respectively. Before discharge from the hospital, children were measured (weight and length), and trained researchers interviewed the mothers. The information collected during the interview comprised socioeconomic and demographic characteristics, mother's health behavior during gestation, and gestational age at childbirth.

Both cohorts were followed-up in 2004/2005 when children were 10/11 years old in RP and 6/7 years old in SL. The follow-up sample was stratified in order to guarantee representativeness of five birth weight groups (<1,500 g; 1,500-2,499 g; 2,500-2,999 g; 3,000-4,249 g; ≥4,250 g). Low birth weight was oversampled by including all low-birth weight children and a random sample of 1/3 of those with normal weight in order to guarantee the power of the study, since the major objective of these cohorts was to study the health of low-birth weight and preterm birth children. After the exclusion of stillborn infants and infants who had died in the first year of life, 1,150 and 926 children were sampled for the follow-up, respectively for RP and SL. Reasons for loss to follow-up in RP and SL included migration, death, and refusal to participate. More detailed information on the methods applied to the cohorts can be found elsewhere (21). The final samples represented 69.4% (n=790) and 72.7% (n=673) of the eligible samples for RP and SL, respectively. The study was approved by the Research Ethics Committee of the Ribeirão Preto Medical School (HC-FMRP-USP; No. 6828/2004) and of the University Hospital, Federal University of Maranhão, Brazil (No. 060/2005).

Information about age (integer years) at first dental visit was obtained during the follow-up interview. Although age at the first dental visit is a discrete variable, competing motivations for this dental visit (prevention *vs* treatment need) results in a non-homogeneous outcome precluding its analysis as such, for example, using specific survival analysis. We were interested in knowing why parents delay dental visits to a point that is unacceptable. Only a small percentage of children had seen a dentist before the age of one and most of them had been to a dentist when 3 to 6 years of age. It is expected that, at the latest, a child will be taken to a dentist at the eruption of the first molars if not before for evaluation of pit and fissure sealant need (22). Therefore, we decided to categorize the variables as <7 and ≥7 years old, a cut-off point indicating the maximum age when a family is expected to take their child to a dentist due to the eruption of the first permanent molar. Moreover, by the age of six (before 7), children are required by law to enter the school system, where parents are usually advised to take their children to a dentist. Our outcome definition also permitted us to compare the two cohorts including censored data (those who had not seen a dentist up to the follow-up interview) in the group aged ≥7 years. Although an additional cut-off point at 3 years of age was suggested and tested, we are presenting only the analysis with a dichotomous variable (<7/≥7) because the final models for both cut-offs were closely similar, except for very large confidence intervals in a multinomial model.

**Figure 1:**
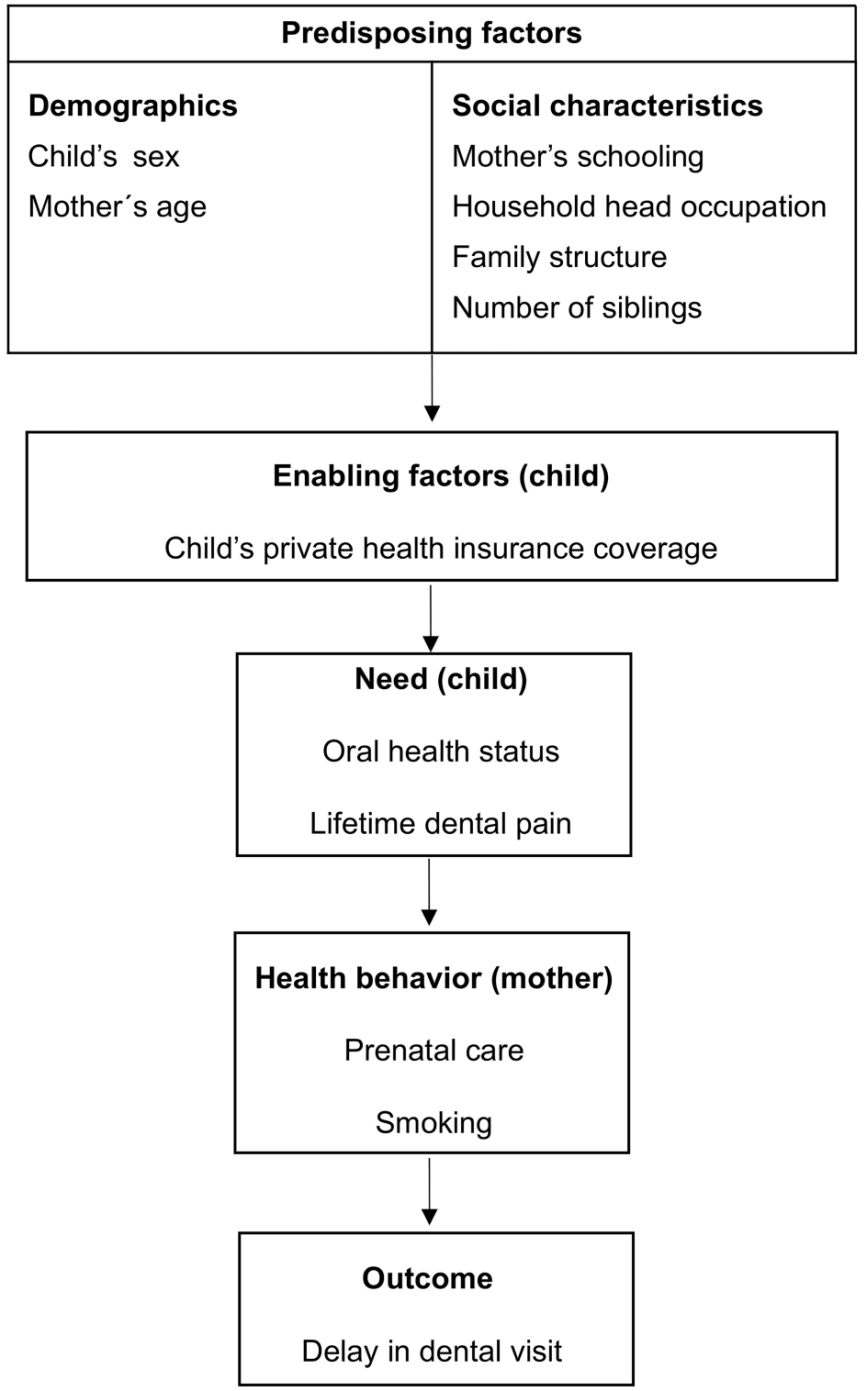
Theoretical model based on the Andersen, Davidson, and Baumeister behavior model of health services utilization (23) .

Information on covariates was obtained from the baseline and follow-up interviews. The explanatory variables included in the study followed a conceptual framework based on the Andersen, Davidson, and Baumeister behavior model of health services utilization (23) (Figure 1). In addition, the model for the choice of variables was guided by the most common variables related to access to health services in the literature. Predisposing factors included demographic (child's skin color and mother's age) and social characteristics of the family (mother's schooling, household occupation, family structure, and number of siblings living in the child's household). As stated by Andersen et al. (23), individual social factors are those “determining the status of a person in the community as well as his ability to cope….[ ]. Traditional measures include education, occupation and ethnicity”. Enabling factors were represented by the continuity of the child's private health insurance. The need factor was represented by child's oral health (measured as perception by the mother) and lifetime dental pain experience due to dental caries. Health behavior factors included mother's number of prenatal care visits and smoking habit during pregnancy. Household head's occupation was categorized as non-manual, skilled manual, and unskilled manual work. Mother's schooling level was also used as a proxy measure for socioeconomic position instead of family income because of the extremely high percentage of parents/caretakers who did not want to report their income. Mothers schooling level was categorized as incomplete primary school and complete primary, middle and secondary schooling, and bachelor's degree. Family structure was classified based on the presence of both biological parents in a nuclear family, a single mother, a mother with a step-parent, a single father with or without a stepmother, and families without biological parents. Child skin color was categorized as white and others because only about 4% of mothers designated their children as being black and most of them reported being of mixed color (mulatto). Continuity of health insurance coverage during childhood was constructed from two questions exploring if the child was covered by private health insurance at the age of three, and also in the previous six months of the follow-up. The two questions were combined, discriminating if the child was covered by private health insurance during one or both periods. Stand-alone private dental insurance in Brazil is not common and most of the time is included at no cost as a complement in general health insurances (24).

### Statistical analysis

Sampling design (stratification and weights) was considered in all statistical analyses, which were performed using the SAS (Version 9.3, SAS Institute, USA) and SUDAAN SAS-Callable (Version 11.0, Research Triangle Institute, USA) statistical packages. Descriptive analyses were followed by stratified analysis (for the understanding of covariate association) and modeling for the estimate of prevalence ratios (PR) using Poisson regression with robust estimation of standard errors (25). Modeling was performed using a hierarchical approach (26). The models were constructed according to the Andersen, Davidson, and Baumeister Behavior Model (Figure 1) beginning with predisposing factors, followed by enabling, need, and health behavior factors. At each level, we kept variables that were significant at the 0.20 level. We kept not only variables that were significant in the model (a=0.05) but also those that were known to be important for health care utilization in order to permit comparison of the two cohorts.

## Results

The information about age at the first dental visit was missing (“don't know”) for 9.7% of children from RP and 19.7% of children from SL. Although no association was significant (Table 1), we observed some trends with an increased proportion of missing data among children living only with their fathers for both cohorts and among children from older mothers and those with less than primary education and with higher education in SL.

**Table 1:** Characteristics of those who did not answer the question about age of the first dental visit in the Ribeirão Preto and São Luís cohorts (2004/2005).

	Ribeirão Preto				São Luís			
	Total	%^*^	SE^†^	P^‡^	Total	%^*^	SE^†^	P^‡^
Gender								
Male	402	10.7	1.7	0.3642	348	18.0	2.2	0.2954
Female	388	8.6	1.5		325	21.4	2.4	
Skin Color^§^								
White	455	10.7	1.6	0.2854	158	23.6	3.5	0.1804
Black/dark skin	335	8.3	1.6		513	18.3	1.8	
Family structure^§^								
Mother & father	545	9.2	1.3	0.5960	346	21.0	2.3	0.8151
Single mother	142	9.0	2.7		165	17.3	3.1	
Mother & stepfather	63	9.2	4.1		60	17.6	4.9	
Single father	20	28.8	11.2		33	23.7	7.8	
Without parents	20	12.8	8.3		67	20.1	5.1	
Oral health^§^								
Excellent	215	11.2	2.3	0.7385	60	25.4	5.8	0.4363
Good	305	9.1	1.8		174	17.1	3.0	
Fair/poor	270	9.2	1.9		439	19.8	2.0	
Lifetime dental pain^§^								
No	540	10.0	1.4	0.7154	292	21.6	2.5	0.2062
Yes	245	9.2	2.0		376	17.5	2.0	
Mother's age								
<20	131	12.0	3.1	0.5662	199	15.8	2.7	0.0786
20-35	563	8.9	1.3		442	20.2	2.0	
≥35	94	11.5	3.5		32	36.8	9.1	
Mother schooling^§^								
<Primary school	62	13.8	4.7	0.8538	42	28.5	7.3	0.2887
Primary school	283	10.2	1.9		181	15.5	2.8	
Middle school	164	8.3	2.4		154	20.2	3.4	
High school	194	8.8	2.3		266	18.2	2.5	
Bachelor degree +	79	8.7	3.2		12	41.4	15.6	
Prenatal care visits								
0-3	215	11.2	2.3	0.7385	60	25.4	5.8	0.4363
4-5	305	9.1	1.8		174	17.1	3.0	
≥6	270	9.2	1.9		439	19.8	2.0	

*Weighted percentage; ^†^standard error; ^‡^P value for Wald test; ^§^information from the follow-up.

**Figure 2:**
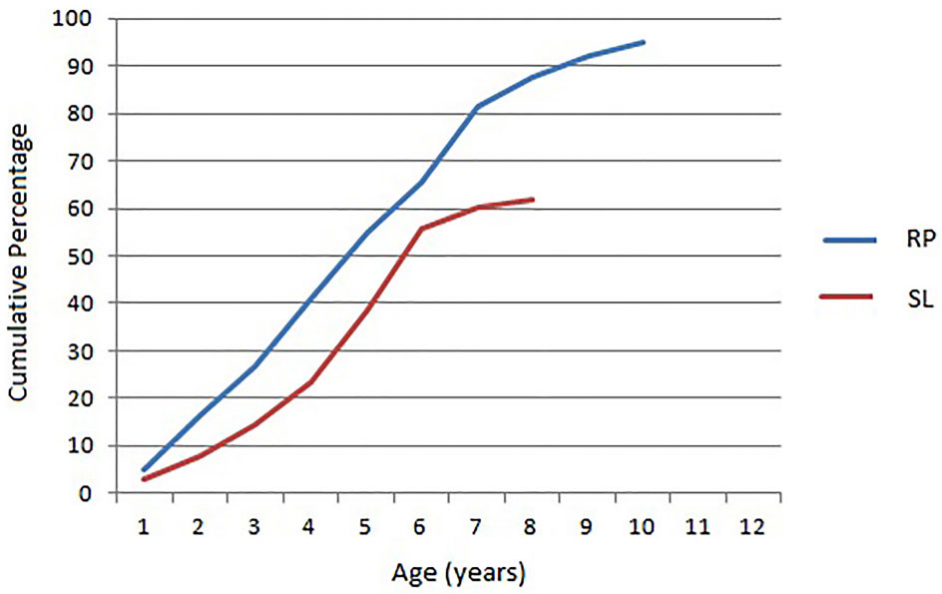
Cumulative distribution of age at first dental visit in the Ribeirão Preto (RP) and São Luís (SL) cohorts (2004/2005). Lines do not add up to 100% because of censored data (those who had not seen a dentist by the time of follow-up).

Figure 2 shows the cumulative distribution of age at the first dental visit. Percentages do not add up to 100% because of censored data, which represented 37.9% for SL and 5.1% for RP. Cumulative percentages were higher for RP than for SL but by the age of six the difference between the two cohorts was smaller (about 10%), showing that 34.5% of children in RP and 44.3% in SL had not seen a dentist by the age of seven.

Bivariate analysis (Table 2) showed that, for both cohorts, the percentage of children not visiting a dentist before the age of seven was significantly higher among those with more siblings, without private health insurance, and lower levels of mother's schooling. For some variables, statistically significant associations were observed only for RP such as being non-white, having poor oral health, and fewer prenatal care visits. Lifetime experience of dental pain was positively associated with not going to the dentist before the age of seven for RP, while it was negatively associated for SL.

Bivariate analysis (Table 2) showed that, for both cohorts, the percentage of children not visiting a dentist before the age of seven was significantly higher among those with more siblings, without private health insurance, and lower levels of mother's schooling. For some variables, statistically significant associations were observed only for RP such as being non-white, having poor oral health, and fewer prenatal care visits. Lifetime experience of dental pain was positively associated with not going to the dentist before the age of seven for RP, while it was negatively associated for SL.

**Table 2:** Bivariate analysis results for children who had not seen a dentist before the age of 7 in the Ribeirão Preto and São Luís cohorts.

	Ribeirão Preto			São Luís		
	n	%^*^ (SE)^†^	P^‡^	n	% (SE)^†^	P^‡^
Mother's schooling						
<Primary school	76	63.1 (5.9)		54	45.5 (6.9)	
Primary school	251	41.9 (3.3)	<0.001	191	46.2 (3.7)	<0.001
Middle school	148	25.2 (3.8)		153	35.7 (4.0)	
Secondary school	93	15.2 (4.0)		132	15.1 (3.1)	
Bachelor's degree +	83	7.7 (3.2)		13	28.0 (12.1)	
Household occupation						
Non-manual worker	138	10.8 (2.8)	<0.001	71	24.1 (5.3)	0.0286
Skilled manual	265	35.9 (3.1)		153	31.2 (3.8)	
Unskilled manual	309	42.9 (3.0)		316	39.1 (2.8)	
Family structure						
Mother & father	495	32.1 (2.2)		274	33.8 (2.9)	
Single mother	131	32.4 (4.3)	0.0769	137	33.4 (4.1)	0.8684
Mother & stepfather	58	56.6 (7.0)		50	37.1 (6.9)	
Single father	14	60.4 (14.1)		26	40.7 (6.7)	
Without parents	17	61.2 (12.3)		54	38.7 (9.8)	
Number of siblings						
0	125	29.9 (4.4)		140	30.3 (4.0)	
1	278	28.1 (2.8)	0.0075	212	28.5 (3.2)	0.0013
2	193	36.2 (3.7)		125	41.0 (4.5)	
3	63	46.1 (6.7)		37	48.5 (8.4)	
4+	56	51.4 (7.0)		29	65.4 (8.9)	
Skin color						
White	388	27.6 (2.4)	0.0004	120	33.9 (4.4)	0.0994
Non-white	324	41.2 (2.9)		421	35.5 (2.4)	
Gender						
Female	360	33.8 (2.7)	0.8365	287	39.3 (4.4)	0.0388
Male	355	34.7 (2.7)		256	30.6 (3.0)	
Health insurance						
Never	234	14.3 (2.5)	<0.001	33	10.2 (5.6)	<0.0001
One period	198	39.4 (3.6)		58	14.1 (4.6)	
Continuous	282	46.2 (3.1)		275	39.4 (2.3)	
Oral Health						
Excellent/very good	191	26.4 (3.4)		45	22.4 (6.2)	
Good	279	31.1 (2.9)	0.0014	145	33.9 (4.0)	0.0988
Fair/poor	245	43.2 (3.4)		353	37.4 (2.6)	
Life-time dental pain						
No	488	29.4 (2.2)	0.0007	354	40.5 (3.3)	0.0281
Yes	222	43.6 (3.5)		309	31.1 (2.7)	
Prenatal care visits^§^						
6+	508	29.5 (2.1)	0.0002	273	31.1 (2.9)	0.0697
4-5	98	41.3 (5.4)		141	35.9 (4.1)	
0-3	42	66.6 (7.9)		117	43.8 (4.7)	
Mother's smoking						
No	581	31.8 (2.1)	0.0122	508	35.3 (2.2)	0.0311
Yes	134	44.7 (4.6)		35	33.8 (8.3)	

*Weighted percentage; ^†^standard error; ^‡^P-value for Wald test; ^§^information from baseline.

Table 3 shows results for each level of the Andersen, Davidson, and Baumeister model (intermediate model) while the final adjusted model is presented in Table 4. Predisposing and enabling factors were very similar in both intermediate (Table 3) and final (Table 4) models. Among predisposing factors, only mother's schooling was statistically associated with a child not going to the dentist before the age of seven for both cohorts. The pattern of association of family structure was similar for both cohorts but only significant for RP. However, for both cohorts, the prevalence ratios for single-father families and families with no biological parents were higher than for nuclear families. The number of siblings was only significant for SL. Having private health insurance, an enabling factor, was negatively associated with dental visit delay for both cities. Among need level, life-time dental pain was statistically associated with dental visit delay for both cities in the intermediate model (Table 3) but remained significant only for SL in the final model (Table 4). The significance of oral health status that was only observed for RP in the intermediate model disappeared in the final model. Among health behavior-related variables, number of prenatal care visits was statistically significant for both cohorts, but only remained significant after adjustment for RP. Smoking that was significant only for RP in the intermediate model lost its significance after adjustment.

**Table 3:** Multivariable analysis for each level of predisposing, enabling, need, and health behavior factors. Results showing prevalence ratios (PR) for covariates for children who did not visit a dentist before the age of 7 in the Ribeirão Preto and São Luís cohorts (2004/2005).

Factors	Ribeirão Preto			São Luís		
	PR	95%CI^†^	P^‡^	PR	95%CI^†^	P^‡^
**Predisposing factors**						
Mother's schooling						
<Primary school	1.00			1.00		0.0019
Primary school	0.67	0.62-1.00	<0.0001	1.03	0.73-1.47	
Middle school	0.43	0.36-0.82		0.92	0.61-1.37	
Secondary school	0.29	0.23-0.88		0.39	0.23-0.68	
Bachelor's degree +	0.20	0.35-0.85		0.71	0.24-2.10	
Household occupation						
Non-manual worker	1.00		0.0894	1.00		0.4020
Skilled manual	1.79	1.00-3.21		1.24	0.76-2.03	
Unskilled manual	1.92	1.07-3.44		1.34	0.86-2.10	
Family structure						
Mother & father	1.00		0.0079	1.00		0.6501
Single mother	0.96	0.71-1.23		1.01	0.75-1.38	
Mother & stepfather	1.22	0.85-1.77		1.04	0.71-1.53	
Single father	1.47	0.86-2.51		1.38	0.90-2.12	
Without parents	2.16	1.37-3.41		1.11	0.66-1.86	
Number of siblings						
0	1.00		0.5965	1.00		0.0057
1	1.24	0.88-1.75		0.93	0.64-1.34	
2	1.25	0.88-1.78		1.31	0.90-1.94	
3	1.44	0.91-2.27		1.39	0.87-2.22	
4+	1.22	0.79-1.87		1.80	1.19-2.73	
Gender						
Female	1.00		0.5894	1.00		0.0846
Male	1.06	0.85-1.33		0.81	0.64-1.03	
Skin color						
White	1.00		0.2842	1.00		0.8633
Non-white	1.14	0.89-1.33		0.98	0.74-1.29	
**Enabling factor**						
Health insurance						
Never	1.00		<0.0001	1.00		<0.0001
For some period	0.85	0.68-1.07		0.36	0.19-0.69	
Continuously	0.52	0.21-0.45		0.26	0.09-0.76	
**Need factors**						
Oral Health						
Excellent/very good	0.88	0.64-1.22	0.0025	0.67	0.37-1.22	0.2000
Good	1.00			1.00		
Fair/poor	1.21	1.02-1.87		1.33	1.00-1.78	
Life-time dental pain						
No	1.00		0.0162	1.00		0.0013
Yes	1.37	1.03-1.69		0.67	0.52-0.85	
**Behavior factors**						
Prenatal care visits^§^						
6+	1.00		<0.0001	1.00		0.0487
4-5	1.38	1.03-1.55		1.16	0.87-1.54	
0-3	2.18	1.66-2.87		1.42	1.09-1.87	
Mother's smoking						
No	1.00		0.0430	1.00		0.4855
Yes	1.31	1.01-1.69		0.82	0.46-1.44	

†95%CI: 95% confidence interval; ^‡^P-value for Wald chi-squared; ^§^information collected at birth.

**Table 4:** Final multivariable analysis results showing prevalence ratios (PR) for covariates for children who did not visit a dentist before the age of 7 in the Ribeirão Preto and São Luís cohorts (2004/2005).

Factors	Ribeirão Preto			São Luís		
	PR	95%CI^†^	P^‡^	PR	95%CI^†^	P^‡^
**Predisposing factors**						
Mother’s schooling^§^						
<Primary school	1.00			1.00		0.0264
Primary school	0.82	0.62-1.00	0.0135	1.09	0.74-1.63	
Middle school	0.54	0.36-0.82		0.99	0.64-1.52	
Secondary school	0.45	0.23-0.88		0.50	0.28-0.89	
Bachelor’s degree +	0.35	0.35-0.85		1.07	0.36-3.16	
Household occupation^§^						
Non-manual worker	1.00		0.2763	1.00		0.7849
Skilled manual	1.61	0.90-2.89		1.11	0.68-1.82	
Unskilled manual	1.52	0.85-2.72		1.16	0.75-1.81	
Family structure^§^						
Mother & father	1.00		0.0004	1.00		0.7401
Single mother	0.90	0.66-1.24		1.06	0.64-1.44	
Mother & stepfather	1.01	0.66-1.53		0.96	0.86-2.07	
Single father	1.77	1.12-2.79		1.33	0.64-1.98	
Without parents	2.34	1.52-3.60		1.12	0.86-2.07	
Number of siblings						
0	1.00		0.7189	1.00		0.0227
1	1.24	0.86-1.78		0.93	0.65-1.33	
2	1.22	0.85-1.77		1.28	0.87-1.88	
3	1.20	0.71-2.04		1.42	0.89-2.07	
4+	1.05	0.67-1.64		1.65	1.09-2.51	
Gender						
Female	1.00		0.2935	1.00		0.0886
Male	1.19	0.89-1.45		0.81	0.64-1.03	
Skin color						
White	1.0		0.2582	1.00		0.9846
Non-white	1.14	0.89-1.47		1.00	0.75-1.33	
**Enabling factor**						
Health insurance						
Never	1.00		0.0391	1.00		0.0227
For some period	1.00	0.77-1.31		0.43	0.22-0.85	
Continuously	0.52	0.32-0.84		0.34	0.12-0.98	
**Need factors**						
Oral health						
Excellent/very good	1.34	0.96-1.86	0.1817	0.75	0.42-1.32	0.2909
Good	1.00			1.00		
Fair/poor	1.21	0.91-1.60		1.14	0.85-1.52	
Life-time dental pain						
No	1.00		0.3761	1.00		0.0014
Yes	1.13	0.86-1.45		0.66	0.52-0.85	
**Behavior factors**						
Prenatal care visits^§^						
6+	1.00		0.0405	1.00		0.5391
4-5	0.91	0.65-1.27		0.90	0.68-1.19	
0-3	1.45	1.05-2.00		1.07	0.81-1.41	
Mother’s smoking						
No	1.00		0.0528	1.00		0.9796
Yes	1.30	1.00-1.69		0.99	0.55-1.78	

†95%CI: 95% confidence interval; ^‡^P-value for Wald chi-squared; ^§^information collected at birth.

## Discussion

In spite of the consensus that children should visit a dentist at the latest by the time of first tooth eruption (1), our study shows that less than 5% of children were taken to a dentist before the age of two in both cohorts. By the age of six, about 34.5 and 44.3% of children in RP and SL, respectively, had not yet visited a dentist and this proportion was still impressively high by the age of eight (RP=12.3% and SL=38.3%). It should be highlighted that this scenario occurred in 2004/2005, i.e., before implementation of the NOHP (11).

Brazil is a country with a health system that is a public-private mixture with free dental services for all individuals through the public Unified Health System (SUS in the Portuguese acronym) (27). However, created in 1988 and with a major expansion after 2003, SUS is still considered to be under development in an attempt to equitably cover the population in collaboration with the private sector (27). A major development of oral health services was observed mostly after 2004 with the NOHP. Among other actions, this policy created a financial incentive for the inclusion of oral health teams in the Family Health Strategy in an attempt to reorganize the Primary Health Care model and to reduce oral health inequalities in Brazil (11). Furthermore, the NOHP promoted the regulation of the Dental Specialty Centers, which was important by increasing access to dental visits for very young children (11). Actually, since our data were collected before the implementation of NOHP in Brazil, it is valid to hypothesize that the current situation should be better, especially in RP, as the provision of oral health services has been considerably widespread within the Family Health Strategy. From 2007 to 2019, there was an expansion of 129% in the proportion of the Brazilian population with access to oral health services in Primary Health Care. However, there are important inequalities among regions and cities in Brazil. In RP, this increase was more than 10 times (from 3.08% in 2007 to 32.57% in 2019), while the coverage remained the same (21%) in SL (28).

Unfortunately, the availability of dental care in the public setting is still a concern, (29) especially for younger children (30). As a result, in spite of a 10% improvement in dental care access compared to the previous decade, two national surveys conducted in 2008 (15) and 2010 (17), respectively, showed that 67.2% of children up to 6 years old and 46.8% of children at 5 years of age had never visited a dentist. Since dental services are also provided by the private sector, it is important to mention the extremely high dentist-to-population ratio in Brazil (1:832) and especially in RP (1:286) and SL (1:568) (31).

When compared to the national average of available information from 2008 (67.2%) (14), our study showed lower percentages of children who had not visited a dentist by the age of seven (35% in RP and 44.3% in SL). By the age of five, the percentages (RP: 45.3% and SL: 54.9%) were also lower than the 63% reported in the Pelotas' population cohort study conducted in the south of Brazil (16). However, our results for 5-year-olds were closer to those of the 2010 National Oral Health Survey (46.8%) (15). The lower percentages observed in our study probably reflect, at least in part, differences in socioeconomic status among our cohorts, the Pelotas' study, and the national average. However, even though RP has one of the highest MHDI in the country, SL and Pelotas have a closely similar low MHDI, and the dentist-to-population ratio in Pelotas (1:471) is even higher than in SL (1:568) (31). Therefore, the reasons for the differences between SL and Pelotas need to be further investigated.

Although the availability of health services is essential for utilization, some individuals are unaware of the benefit or unable/unwilling to use the services even when they are provided free of charge (32). Delay in dental visits can also be observed in well-established universal health systems such as that of the United Kingdom, where the rate of dental visits by the age of two has been stable around 30%. A low percentage of dental visits by the age of two (44.5%) was also reported in the evaluation of the Free First Visit program in Canada (33) which, however, did not consider the non-take up of the program (32). More recently, timing of first dental visit has only been reported in studies from convenience samples such as one study in India, where 57% of children seen at the pediatric dentistry department of a tertiary-care hospital in Puducherry had their first dental visit by the age of 6-9 years (34). Another study in Poland showed that 92.2% of the children who went for the first time to four private dental clinics had their first visit before 7 years of age (35). However, due to the sampling design, those results are subject to selection bias, overestimating prevalence.

In our study, even among the most highly educated mothers, only 39.9% in RP and 9.73% in SL had taken their children to a dentist by the age of two. It would be expected that highly educated mothers would be able to afford dental services or have the means to access those that are free of charge. A possible explanation is that these mothers consciously postpone the first dental visit because of a lower prevalence of dental caries among their children. Moreover, we should take into account beliefs and low literacy regarding the importance of children's oral health (36).

Comparison of the cumulative percentage of children who had not visited a dentist showed that the difference between the two cities was smaller around the age of six (a 10% difference). This decrease in the difference might be related to the reasons for taking children to their first dental visit, which might be the eruption of the first permanent molars and the enrollment in elementary school, both taking place around the age of six. However, the SL cohort showed lower cumulative levels for children younger than six and especially for children older than seven. Although we do not have information about the availability of dental services at public community centers for RP children, the dentist-to-population ratio for the RP population is considerably higher (1:286) than that for SL (1:568), a fact that may explain in part these differences (31).

Multivariable analysis revealed that mother's schooling and private health insurance were the only variables associated with not visiting a dentist before the age of seven for both cohorts. Although the association of health insurance and health service utilization is well known, we should be careful with our interpretation. As stated before, private dental insurance is not common in Brazil and most of it is offered in combination with general health insurance (24). Therefore, in this study, the association with health insurance is probably more related to the purchasing power of the family than specifically to the access to dental services. Moreover, since the variable we used in this study described the continuity of health insurance coverage throughout the child's life, this variable probably also measures the economic stability of the family, which would more likely result in health-seeking behavior.

The contrasts in the final models for RP and SL probably reflect differences in socioeconomic structure, as well as in the prevalence and distribution of dental caries among the cohorts. Although we did not have information on dental caries from dental examinations, our study showed that 65.3% of mothers reported poor oral health for their children in SL compared to 34.7% in RP. Moreover, although poor oral health was associated with mother's schooling in both SL and RP, it was more widespread in SL than in RP. In the SL cohort, poor oral health of the child was reported by 71.6% of mothers of lower educational level compared to 61.5% of mothers with the highest educational level. In contrast, these percentages were respectively 53.6 and 12.0% in RP. Moreover, lifetime dental pain due to dental caries was significantly higher among SL children (56.4%) already at 7/8 years of age compared to RP children at 11/12 years of age (32.4%). Taking this evidence into consideration, we may infer that the negative association of lifetime dental pain with not visiting a dentist before the age of seven in SL was probably a result of health care-seeking behavior. It is important to point out that this association found in SL was not a result of over-adjustment by oral health perception.

Although family structure is an important determinant of children's health care utilization (37 [Bibr B38]), there are only few studies addressing its association with dental services (37–39). In our study, we detected an increased prevalence of children who had not visited a dentist before the age of seven among those living in single-father families and in families with no biological parents compared with those living in nuclear families. We found only one study in which single-father families were studied and were associated with lower rates of child health service utilization in general, except for dental service utilization, which was explained by socioeconomic variables (39). In contrast to the literature reporting lower health service utilization among children from single-mother families, we could not observe this trend in either cohort.

Two health behaviors related to mothers, i.e., low number of prenatal care visits and smoking habit, were associated with a child not visiting a dentist before the age of seven only in RP. The lack of association with mother smoking habit in SL might be explained by the well-known low prevalence of smoking among women from SL (40). Using the same reasoning, a very high prevalence of a type of exposure might also turn into a weak indicator of an event, as might have been the case for the high percentage (50%) of low number of prenatal visits in SL. Similarly, a large number of siblings was an important factor associated only in the SL cohort. The lack of significance for number of siblings in RP might be explained by the strong association between the outcome of interest and mother's educational level which, when added to the model, removed the significance of number of siblings as a predictor.

Among the limitations of this study is the possibility of recall bias related to the outcome, which was reported at follow-up. However, the first dental visit is frequently a special event in a child's life, requiring mothers to make an appointment either for preventive reasons or because of pain or trauma, which might limit recall bias. The lack of information about the motives of the first dental visit may be claimed as another potential limitation. However, as discussed before, the reasons for taking a child to a dentist for the first time (treatment vs prevention) may compete with each other, and our major objective was to characterize those children who had not seen a dentist before the age of seven, which would be considered unacceptable. It is important also to point out that the data used in this study were collected in 2004/2005, before the NOHP was implemented. Probably, there has been some improvement in access to oral health services; however, no new population-based study reporting delay in dental visits for Brazilian children has been published since 2013. The present data are therefore important for future comparisons and analysis of the long-term effect of the NOHP.

In spite of its limitations, this study had several strengths. It is the first report of timing of the first dental visit in two population cohorts using the same research protocol, which included several important associated factors collected at birth and at follow-up. This enabled us to study the same outcome with similar availability of dental care in a heterogeneous population with a lower prevalence of dental caries polarized among the poorest (RP) compared to a more homogeneous population with high prevalence and widespread distribution of dental caries (SL).

In conclusion, this study revealed that, in 2004-2005, a very low percentage of children of both cohorts were taken to their first dental visit within the recommended period of 12 months of age. Moreover, delay in dental visits after the age of six was very high even among children of more educated mothers. Further studies are necessary to analyze what has changed in access to the first dental visit after the NOHP in Brazil and to understand what underlies the decision of Brazilian mothers to take their children to the dentist for the first time.
